# Supplementary figures and images for: Genome Scan of Rice Landrace Populations Collected Across Time Revealed Climate Changes’ Selective Footprints in the Genes Network Regulating Flowering Time
Source: Rice (N Y). 2023 Mar 22;16:15. doi: 10.1186/s12284-023-00633-4 (PMC10033818; doi:10.1186/s12284-023-00633-4)

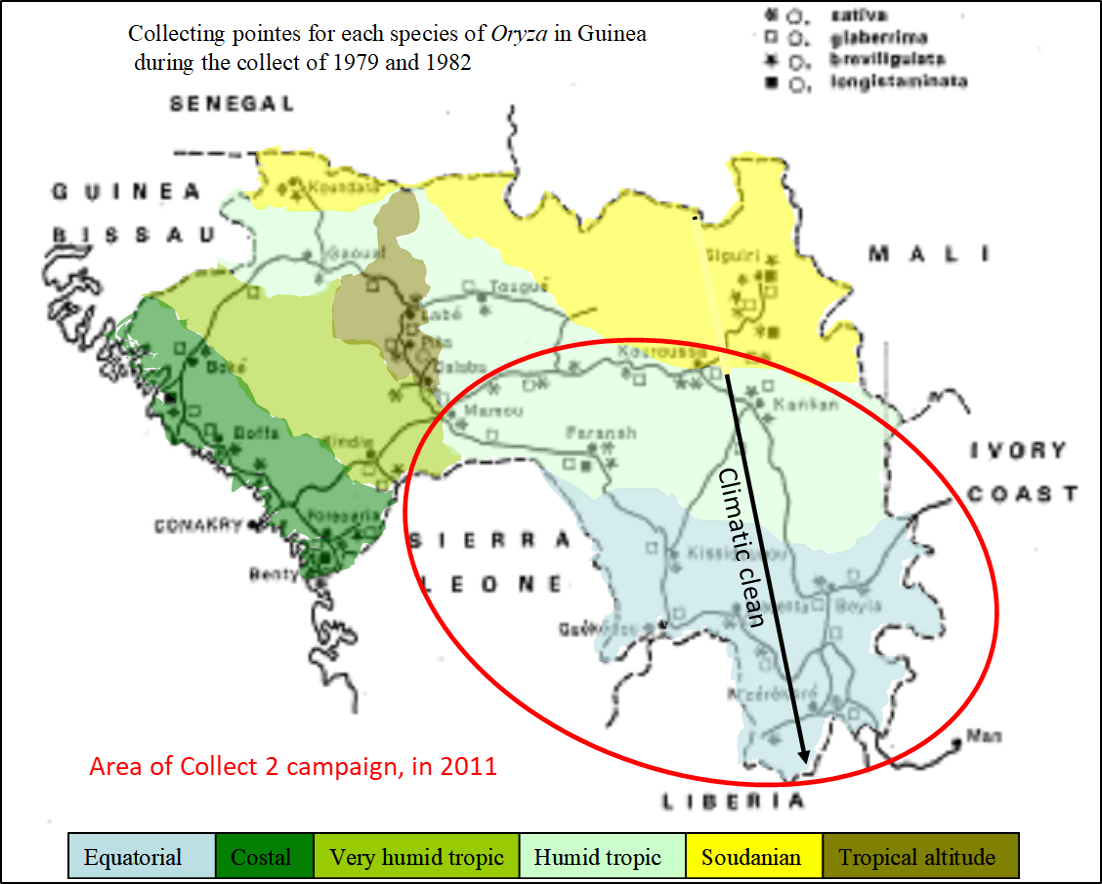

Supplement: Supplementary file 1 — Additional file 1: Fig. S1. Area and road map of the two collect campaigns of rice samples in Guinea. Adapted from Bezançon et al. (1983). [file 12284_2023_633_MOESM1_ESM.tif]

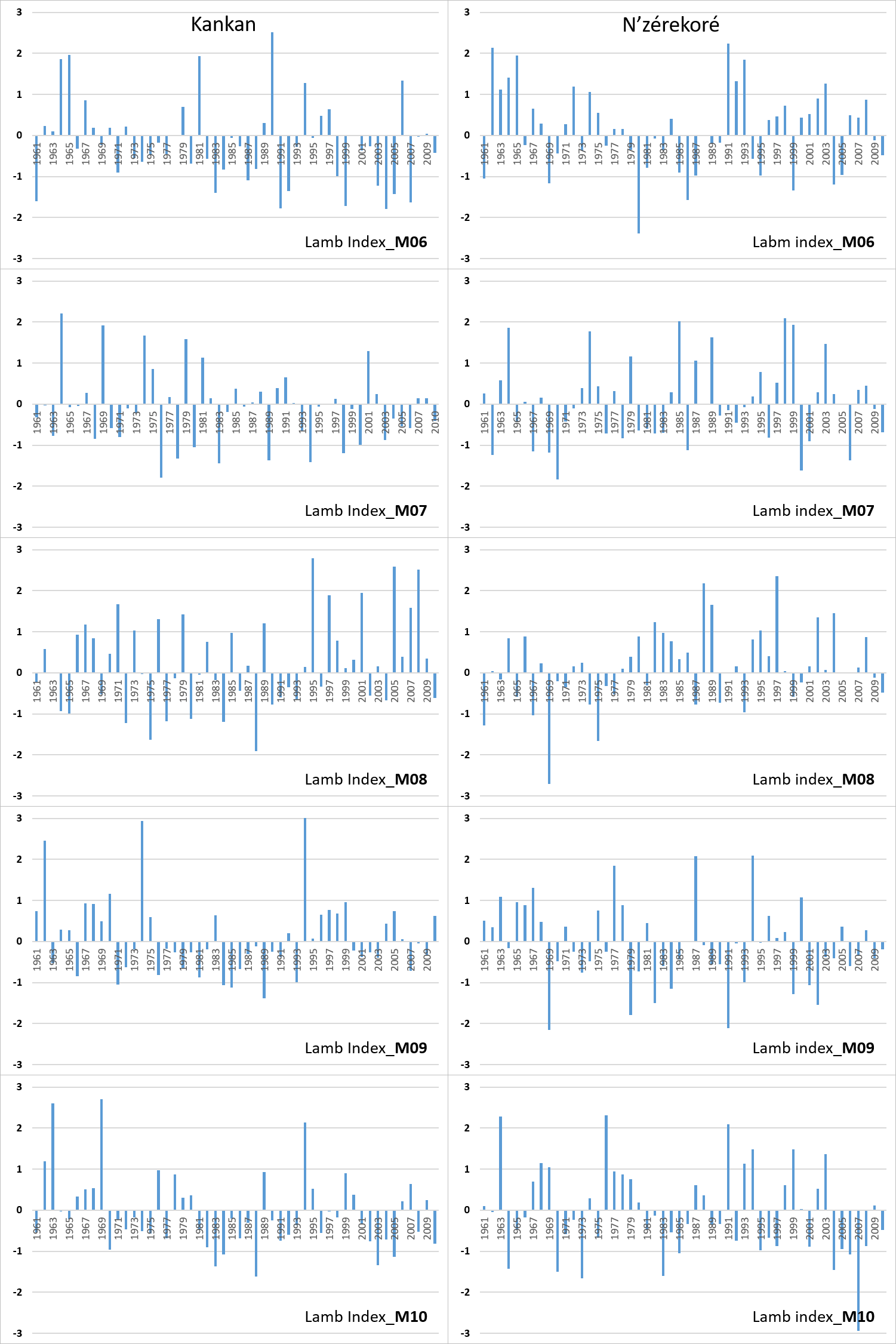

Supplement: Supplementary file 2 — Additional file 2: Fig. 2. Pattern of deviation of the monthly rainfall during crop growing season (June–October), from the normal reference, during the 1961–2010 period, in Kankan (10°23′01.65″N, 9°18′18.72″W) and N’Zérekoré (7°48′53.2″N, 8°42′14.11″W) sites of Guinea. [file 12284_2023_633_MOESM2_ESM.tif]

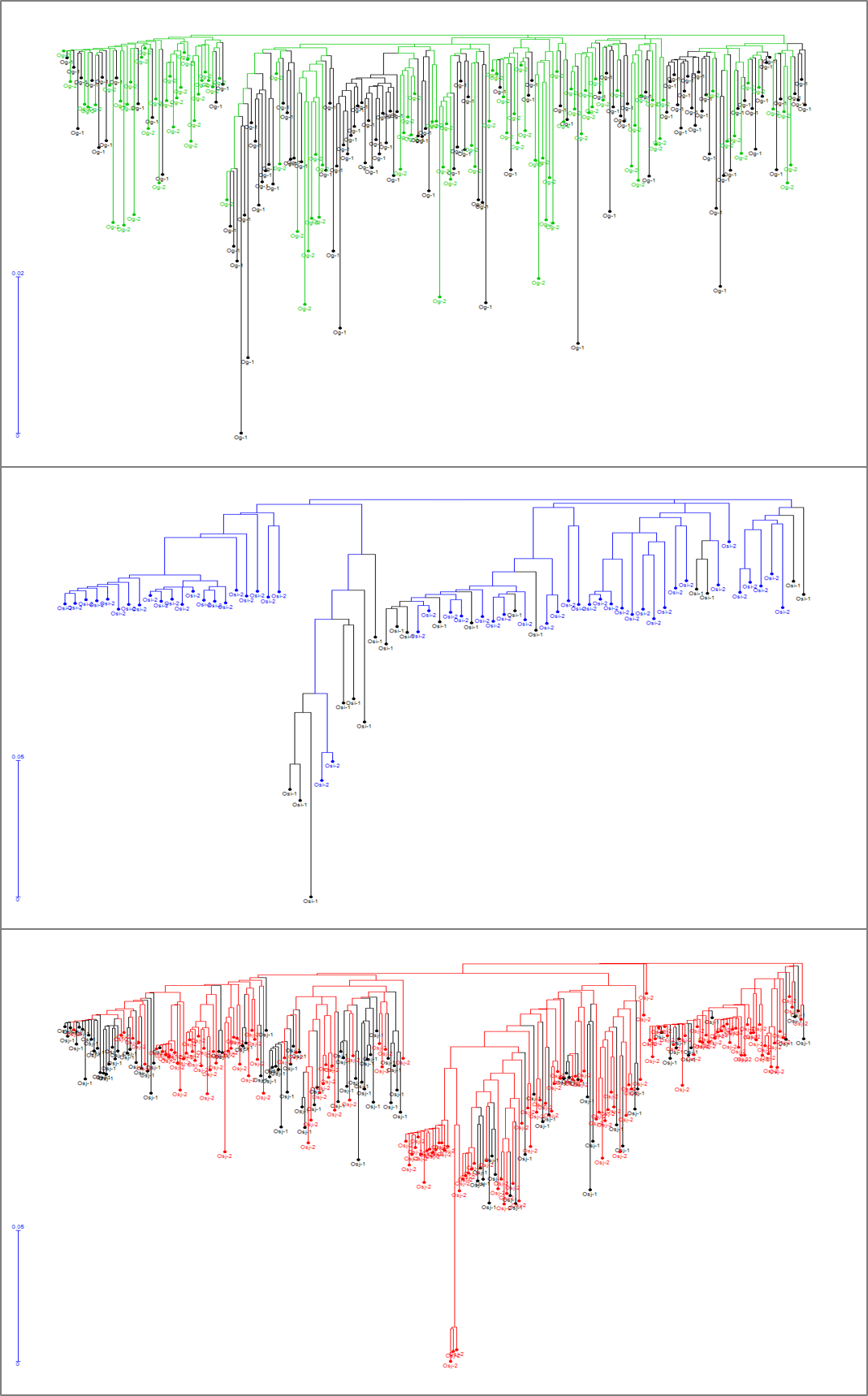

Supplement: Supplementary file 4 — Additional file 4: Fig. S3. Unweighted neighbor-joining tree of simple matching distances constructed from genotypes at 1.130 SNP loci, for O. glaberrima (Og) O. sativa indica (Osi) and O. sativa japonica (Osj) groups. Accessions from the first collect time (Og-1, Osi-1 and Osj-1) are shown in black. [file 12284_2023_633_MOESM4_ESM.tif]

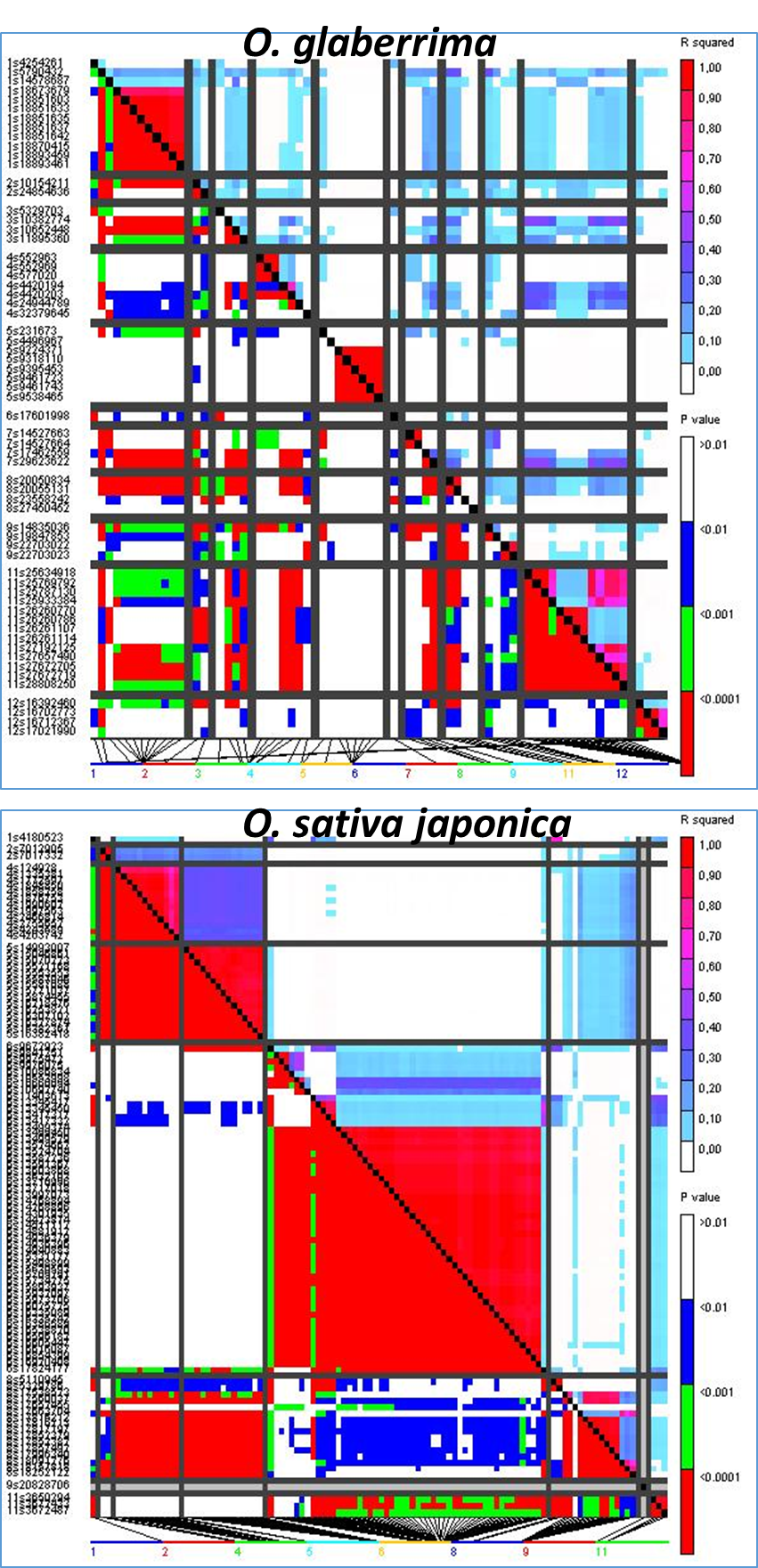

Supplement: Supplementary file 8 — Additional file 8: Figure S4. Linkage disequilibrium between SNP loci under selection in O. glaberrima and O. sativa japonica (74 and 110 SNP loci respectively). Triangle above and below the bisectrix represent the r² and the r² p value respectively. [file 12284_2023_633_MOESM8_ESM.tif]
